# Reply to Pirrone and Tsetsos: Robust evidence for enhanced high-value sensitivity

**DOI:** 10.1073/pnas.2209521119

**Published:** 2022-08-15

**Authors:** Blair R. K. Shevlin, Stephanie M. Smith, Jan Hausfeld, Ian Krajbich

**Affiliations:** ^a^Department of Psychology, The Ohio State University, Columbus, OH 43210;; ^b^Booth School of Business, University of Chicago, Chicago, IL 60637;; ^c^Faculty of Economics and Business, University of Amsterdam, 1018 WB Amsterdam, The Netherlands;; ^d^Department of Economics, The Ohio State University, Columbus, OH 43210

Pirrone and Tsetsos (1) offer an insightful perspective on our article in PNAS (2), where we show that people are better at discriminating between high-value options. Pirrone and Tsetsos argue that value representations and decision computations are distinct, and that for high-value options there may be heightened motivation rather than increased value sensitivity. They also argue that our experimental design may have cued changes in motivation.

We agree with Pirrone and Tsetsos (1) that valuation and decision computations are distinct. Neuroimaging work suggests that reward regions compute subjective values, which are then integrated into decision values in other, mostly frontal regions (3). While there is evidence for decreasing sensitivity among reward-related neurons in the parietal lobe (4), whether these neurons represent value or numeric information is debatable (5). Nonetheless, Pirrone and Tsetsos speculate that decreasing value sensitivity in reward circuity is compensated for downstream by increasingly motivated decision computations.

In our article, we were cautious not to attribute behavior to unobserved internal processes. Our primary interest was behavior. Here, the distinction between our model of increasing value sensitivity and Pirrone and Tsetsos’s (1) model of decreasing value sensitivity plus heightened motivation is arguably irrelevant, since the behavioral implications are identical. Our results indicate that the function that maps value inputs to choices is convex, even if there may be intermediate concave transformations.

Pirrone and Tsetsos’s (1) hypothesis also fails to explain why there should be increased motivation for high-value decisions. In our experiments, the difficulty was matched so that the benefits of making accurate decisions were equivalent across value levels. Furthermore, we did not instruct participants how to behave at different value levels. Stating that people are more motivated to make high-value decisions takes for granted the phenomenon that we observed in the first place.

Setting aside the issue of why, there is also the issue of how high-value options could motivate decision makers. Pirrone and Tsetsos (1) argue that our blocked experimental design heightened our participants’ awareness of overall value and gave them more exposure to high-value items. However, there were equal numbers of exclusive-value and mixed-value blocks, and their order was randomized. Moreover, participants spent less total time on high-value trials (high value: M=209.90 s, SE=10.11; middle value: M=216.38 s,SE=10.45; low value: M=222.06 s,SE=10.50). In a follow-up experiment (*n* = 52) (6), using the stimuli from Study 3 (2) and only mixed-value blocks, we again find that, at higher values, participants are more accurate (mixed-effects logistic regression of accuracy on overall value, controlling for absolute value difference and absolute variability difference: p=0.027) and faster (mixed-effects regression of log(RT) on overall value, controlling for the same variables: p<0.001). Thus, we are confident that increasing value sensitivity is not a product of our original studies’ blocked designs.

In conclusion, our data provide an important challenge to decreasing value sensitivity in choice behavior. However, we agree with Pirrone and Tsetsos (1) that our work does not directly address neural value representation. We hope that future research can help to clarify the link between value representation and behavior.
